# Quantification of deoxythioguanosine in human DNA with LC-MS/MS, a marker for thiopurine therapy optimisation

**DOI:** 10.1007/s00216-024-05581-6

**Published:** 2024-10-13

**Authors:** Björn Carlsson, Louise Karlsson, Andreas Ärlemalm, Sophie Sund, Malin Lindqvist Appell

**Affiliations:** 1 Department of Clinical Pharmacology and Department of Biomedical and Clinical Sciences, Linköping University, Linköping, Sweden; 2 Department of Biomedical and Clinical Sciences, Linköping University, Linköping, Sweden

**Keywords:** Thiopurine, DNA, LC-MS/MS, 2′-Deoxythioguanosine, Therapeutic drug monitoring

## Abstract

In the treatment of diseases such as acute childhood leukaemia (ALL) and inflammatory bowel disease (IBD), the thiopurines azathioprine, 6-mercaptopurine, and 6-thioguanine are used. Thiopurines are antimetabolites and immunomodulators used to maintain remission in patients. They are all prodrugs and must be converted into the competing antimetabolites thioguanosine triphosphate and deoxythioguanosine triphosphate for final incorporation into RNA or DNA. The current therapeutic drug monitoring (TDM) method measures the sum of the formed metabolites in the sample, after acidic hydrolysis at high temperature. In this work, the goal is to measure these drugs closer to their pharmacological endpoints, once incorporated into DNA. After extracting DNA from whole blood, followed by DNA hydrolysis, 2′-deoxythioguanosine (dTG) and the complementary natural nucleobase 2′-deoxycytidine (dC) were measured. Chromatographic separation on a HSS T3 column followed by mass spectrometric detection was performed in multi-reaction monitoring (MRM) mode on a Xevo TQ-XS with ESI in positive mode, within 5 min. The concentration range for dTG was 0.04–5 nmol/L, and for dC, 0.1–12.5 µmol/L. The lower limit of detection was determined to a concentration of 0.003 nmol/L for dTG and 0.019 µmol/L for dC. The intra- and inter-assay imprecision for the quality controls ranged between 3.0 and 5.1% and between 8.4 and 10.9%, respectively. Sample stability for up to 4 years is shown. In summary, a sensitive method to quantify the thiopurines incorporated into DNA as dTG has been developed and will be used in further clinical studies for a better understanding of the mode of action of the thiopurines and the use of this method in TDM.

## Introduction

**Fig. 1:**
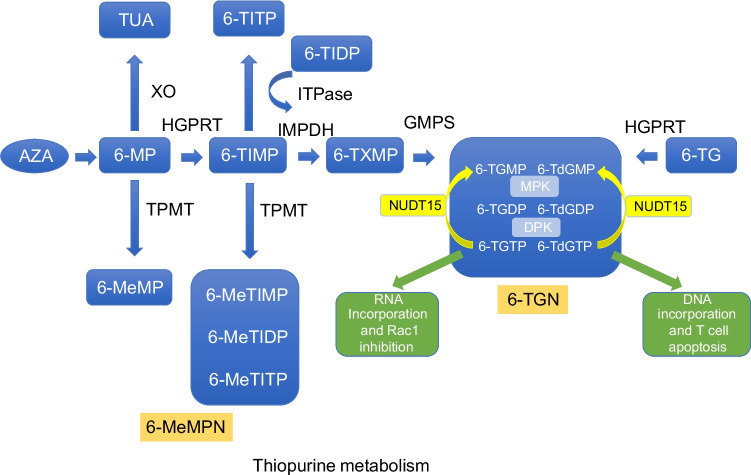
Thiopurine metabolism. Drugs: AZA, azathioprine; 6-TG, 6-thioguanine; 6-MP, 6-mercaptopurine. Metabolites: TUA, thiouric acid; 6-MeMP, 6-methylmercaptopurine; 6-TIMP, 6-TIDP, 6-TITP, thioinosine mono/di/triphosphate; 6-MeTIMP, 6-MeTIDP, 6-MeTITP, methylthioinosine mono/di/triphosphate (6-MeMPN); 6-TXMP, thioxantine monophosphate; 6-TGMP, 6-TGDP, 6-TGTP, thioguanosine mono/di/triphosphate; 6-TdGMP, 6-TdGDP, 6-TdGTP, deoxythioguanosine mono/di/triphosphate (6-TGN). Enzymes: XO, xanthin oxidase/dehydrogenase; TPMT, thiopurine methyltransferase; HGPRT, hypoxanthine-guanine phosphoribosyl transferase; ITPase, inosine triphosphatase; IMPDH, inosine-5′-monophosphate dehydrogenase; GMPS, guanosine monophosphate synthetase; MPK, monophosphate kinase; DPK, diphosphate kinase; NUDT15, nudix hydrolase 15

Thiopurines such as azathioprine (AZA), 6-mercaptopurine (6-MP), and 6-thioguanine (6-TG) are drugs that are used for treatment of chronic inflammatory autoimmune disease and leukaemia, and to prevent rejection after organ transplant [1, 2]. The pharmacology around these drugs is complex and there are large inter- and intra-individual variations. Thiopurines are all prodrugs without any intrinsic biological activity and require conversion, through various steps, to their active metabolites intercellular 6-thioguanine nucleotides (6-TGN) and 6-methylmercaptopurine nucleotides (6-MeMPN). The active metabolites affect fast-growing cells, with an immunosuppressive effect on the immune system and a cytotoxic effect on cancer cells [3, 4]. AZA is converted to 6-MP mainly in the liver to the active metabolites 6-TGN by three different ways mediated by inosine-5′-monophosphate dehydrogenase (IMPDH), thiopurine methyl transferase (TPMT), hypoxanthine-guanine phosphoribosyl transferase (HGPRT), and xanthin oxidase/dehydrogenase (XO) in the intestinal wall (Fig. 1). TPMT and XO convert 6-MP into the inactive metabolites 6-methylmercaptopurine (6-MeMP) and thiouric acid (TUA). Hypoxanthine-guanine phosphoribosyl transferase (HGPRT) converts 6-MP to thioinosine monophosphate (6-TIMP). 6-TIMP is then further converted in three different ways, to the methylated form 6-meTIMP, 6-meTIDP, and/or 6-meTITP, the 6-MeMPN, by TPMT or by IMPDH into thioxantine monophosphate (6-TXMP), which is further metabolised into the active metabolites 6-TGMP, 6-TGDP, 6-TGTP (with ribose), 6-TdGMP, 6-TdGDP, and 6-TdGTP (with deoxyribose), commonly called the 6-TGN. 6-TGMP/6-TdGMP is phosphorylated to the diphosphate (6-TGDP/6-TdGDP) by monophosphate kinase (MPK) and further to the triphosphate 6-TGTP/6-TdGTP by diphosphate kinase (DPK). Finally, inosine triphosphate pyrophosphatase (ITPase) can convert 6-TITP back to 6-TIMP. Completing the destination for the final pharmacological effect is the incorporation of 6-TdGTP into DNA and 6-TGTP into RNA, with nucleotide suppression, subsequent mismatching, and cell apoptosis (Fig. 1). TPMT is a polymorphic enzyme; accordingly, loss-of-function variants of TPMT are strongly associated with thiopurine-induced severe leukopenia [5]. Around 9% of the Caucasian population carries one loss-of-function allele and 1 in 300 carries two non-functional alleles, leading to undetectable activity [6], increasing the risk of toxicity when treated with conventional thiopurine doses. In some populations, including Asians, nudix hydrolase 15 (NUDT15) genetic variants are more common than TPMT variants, making this a significant biomarker for toxicity. NUDT15 converts metabolites 6-TGTP to 6-TGMP and 6-TdGTP to 6-TdGMP (Fig. 1). The genetic variant, c.415C>T, R139C results in an instable enzyme that finally increases the incorporation of 6-TGTP and 6-TdGDP into RNA and DNA, resulting in leukopenia [7, 8].

The combination of TPMT geno- and/or phenotyping, together with thiopurine metabolite monitoring to individualise thiopurine treatment by measuring the concentration of the metabolites in erythrocytes or in whole blood, is fundamental for therapeutic drug monitoring (TDM). HPLC with UV detection or mass spectrometric detection are the most-used methods for measuring thiopurine metabolites, in which the methods by Lennard et al. and Derviuex et al. have been the cornerstones [9–12]. These methods differ in the extraction procedure, but for both methods, the 6-TGN and 6-MeMPN are hydrolysed to 6-thioguanine (6-TG) and 4-amino-5-amino-5-(methylthio)carbonyl imidazole (AMTCI) for the methylated thioinosine derivate. 6-TG is the sum of 6-TGN, 6-thioguanosine, and 6-TGN, including their deoxynucleotides, while AMTCI is the sum of 6-MeMPN and 6-MeMP, including their corresponding deoxynucleotides (see Fig. 1). It is possible to measure the individual metabolites, but studies demonstrate no added value for TDM compared to the methods by Lennard and Dervieux [13, 14].

Measuring therapeutic response closer to the pharmacological endpoint, incorporation into DNA, with determination of 2′-deoxythioguanosine (dTG), may be a more relevant marker, in combination with earlier methods, with the object of improved treatment outcome [2, 15]. Previously, analytical methods for determination of dTG in leukocyte`s DNA have, after hydrolysis with or without derivatisation, utilised HPLC with fluorescence or mass spectrometric detection [16–20]. Both a TDM based on dose-concentration–controlled thiopurine therapy and a measurement located at the effect site at the DNA level have shown an association between incorporated dTG levels and relapse risk in ALL patients [21]. Initial studies on IBD patients and dTG incorporation have so far shown promising results; however, these studies were performed on smaller patient cohorts [22]. In our study, a major cohort of IBD patients, both already on or eligible for thiopurine medication, will further scrutinise whether dTG is a better or a complementary measure for IBD patients.

The aim of this study was to develop and validate a sensitive, quantitative, and robust LC-MS/MS method for measuring incorporated dTG and the complementary nucleobase 2′-deoxycytidine (dC) in DNA from nucleated blood cells.

## Material and methods

### Chemicals and reagents

Analytical standards of dTG, dC, dA (2′-deoxyadenosine), dT (2′-deoxythymidine), and dG (2′-deoxyguanosine) were purchased from Sigma-Aldrich (St. Louis, USA); the internal standards 2′-deoxycytidine-^13^C^15^N_2_ (dC-IS) and 2′-deoxyguanosine-^13^C_10_^15^N_5_ (dTG-IS) from Toronto Research Chemicals (Ontario, Canada). Purity levels for the analytical and internal standards were, for dTG, 98.4%; for dC, 100%; for dA, 100%; for dG, 99%; for dC-IS, 97%; and for dTG-IS, 93%. Water was purified with a Milli-Q gradient water purifying system (Merck, Darmstadt, Germany). Methanol (LCMS grade) and formic acid (EMSURE®) were from Merck; dimethyl sulfoxide (DMSO) (BioUltra) and dithiothreitol (DTT) were from Sigma-Aldrich (St. Louis, USA). DNA extraction was done on a Maxwell 16 with Maxwell® 16 blood DNA purification kit AS1010 (Promega Corporation, Madison, WI, USA). DNA concentration measurement was performed with a NanoDrop 1000 (Thermo Fischer Scientific, Waltham, USA). For DNA hydrolysis, the EpiQuik™ one-step DNA hydrolysis kit P-1023-6 (Epigentek Group Inc., Farmidale NY, USA) was used [23]. For method development and validation purpose, blank human blood from healthy human volunteers at Linköping University Hospital was used and approved by the Medical Ethics Committee.

### Standards and quality control samples

Preparation of stock solution, 1 mg/mL, was made in DMSO for dTG, while the natural nucleobases dA, dC, dT, and dG, were prepared in ultrapure water and stored at −20°C. Stock solutions of 1 mg/mL dC-IS and dTG-IS were prepared in methanol and stored at −20°C. Working solutions of dTG and dC, for calibrators, and for quality controls (QCs) were made in 1 mM DTT. An eight-point calibration curve was prepared by further dilution in 1 mmol/L DTT with a final highest concentration of 5 nmol/L for dTG, followed by serial dilution to obtain the concentration of 2.5, 1.3, 0.6, 0.3, 0.16, 0.08, and 0.04 nmol/L and 12.5 µmol/L for dC with serial dilution to 6.3, 3.1, 1.6, 0.8, 0.4, 0.2, and 0.1 µmol/L, when added to the hydrolysis buffer mix. QC samples were prepared by using separately prepared stock solutions by adding the dTG and dC to 1 mmol/L DTT to make the highest QC-III with dTG of 3.75 nmol/L and dC 9.4 µmol/L, and further dilution in 1 mmol/L DTT to QC-II with dTG of 0.5 nmol/L and dC 1.3 µmol/L and QC-I with dTG of 0.1 nmol/L and dC 0.25 µmol/L of each analyte, when added to the hydrolysis buffer mix. An internal standard working solution was prepared in 10.8 nmol/L DTT with dTG-IS at a concentration of 11 nmol/L and dC-IS 27.5 µmol/L.

### Sample collection

From patients with IBD, whole blood samples were collected in EDTA tubes. These patients, in an ongoing study, had been treated on different doses of AZA and were deemed to be in clinical remission. The whole blood samples were aliquoted for further processing and stability studies and stored at −80°C. Whole blood from healthy donors was extracted to obtain blank DNA without any detectable dTG, and stored at −80°C.

### Sample preparation

**Fig. 2:**
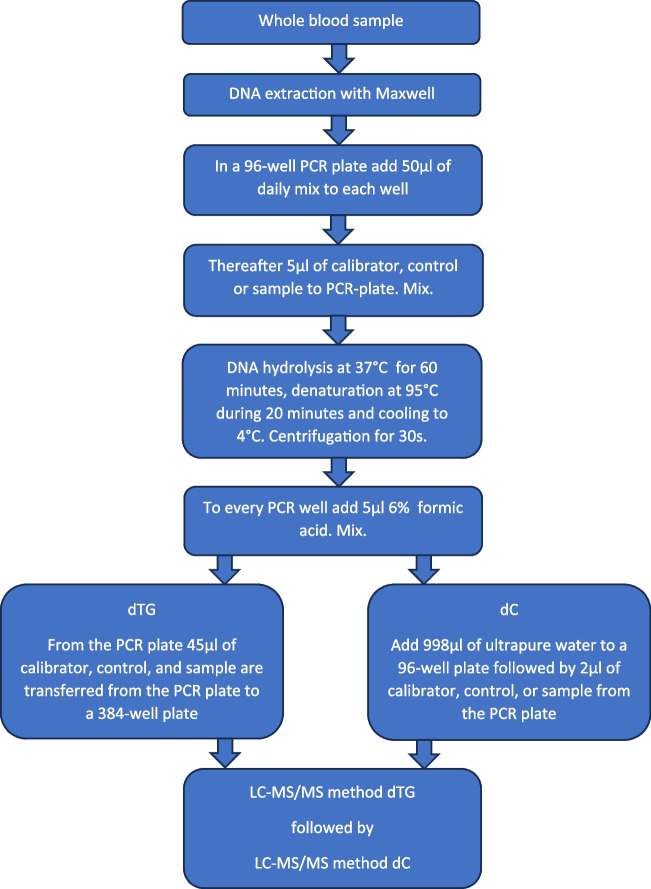
Sample preparation procedure

The procedure for the sample preparation begins with DNA extraction from the collected whole blood sample with a Maxwell 16 DNA purification kit according to the manufacturer’s instructions. The extracts containing DNA were aliquoted into two 100-µL portions and stored at −80°C. One portion of 10 µL was used for quantification of the amount of DNA in the extract by using a NanoDrop 1000 spectrophotometer. For the LC-MS/MS analysis, samples were thawed and processed in batches of 50. The thawed DNA patient samples were digested into nucleosides by using the EpiQuik™ one-step DNA hydrolysis kit. Calibrators and controls were also treated with EpiQuik™. At first, a fresh daily mix of the EpiQuik reagents and internal standard working solution was made. With a repetitive dispensing pipette, 50 µL of the daily mix was added to the wells in a 96-well, 0.2-mL, PCR plate. Afterwards, 5 µL of calibrator, controls, or patient samples was added to the wells and finally mixed with a multichannel pipette. After this, the hydrolysis was performed on a Veriti thermal cycler (Applied Biosystems, Foster City, CA). The following program cycle was used: DNA hydrolysis at 37°C for 60 min, denaturation of hydrolysed enzymes at 95°C for 20 min and cooling to 4°C. When completed, the PCR plate was centrifuged. Ultimately, using an epMotion 5075 Liquid handler (Eppendorf, Hamburg, Germany), the samples for dTG measurement were transferred to a 384-well plate and the samples for dC were diluted with ultrapure water in a 96-well plate before injection on the LC-MS/MS system (see the schematic procedure in Fig. 2). Two separate injection methods were used, with sets of calibrators, controls, and samples. One set for dTG and one set for dC were injected.

### Chromatography and instrumentation

LC-ESI-MS/MS analysis was performed on an Acquity UPLC I-Class system (Waters, Milford, USA) equipped with a binary solvent manager, sample organiser, autosampler with a 5-µL loop, and a column manager connected to a Xevo TQ-XS tandem quadrupole (Waters). Chromatographic separation was performed on an Acquity HSS T3 150 × 2.1 (150 × 2.1 mm, 100 Å pore size, 1.8 µm particle size) preceded by a pre-column, VanGuard Acquity HSS T3 (5 × 2.1 mm, 100 Å pore size, 1.8 µm particle size), all from Waters. The column compartment was held at a temperature of 45 °C. The autosampler weak needle wash was 10:90 acetonitrile:water and the strong wash was 80:20 acetonitrile:water. Sample injection mode was a full loop, at 5 µL. The four natural nucleobases—dTG and their internal standards for dTG and dC—were separated in a gradient chromatographic run followed by positive mass spectrometric detection. The mobile phase consisted of 0.1% formic acid in water (A) and 100% methanol (B) at a flow rate of 0.5 mL/min over the total runtime of 5 min. The time program for the gradient separation was as follows: phase B was started at 9% for 4 min; then increased to 90% from 4.0 to 4.2 min; and at 4.5 min, was returned to 9% and equilibrated until 5.0 min.

The mass spectrometer was operated in positive ion mode. The instrument conditions were as follows: capillary voltage 0.8 kV, desolvation gas flow and temperature were maintained at 1000 L/h and 500°C, respectively, the source temperature was set to 150°C, and the sample cone gas flow was set to 150 L/h and collision gas (argon) at 0.15 mL/min.

All analytes and internal standards were detected using multiple reaction monitoring (MRM) recording one quantifier ion, one qualifier ion for each of the analytes, and a quantifier ion for the internal standards. The specific mass spectrometric settings for each compound are presented in Table 1. An eight-level calibration curve was generated before every analysis, by calculating the area ratios of dTG/dTG-IS and dC/dC-IS to their corresponding concentrations, with a linear regression model, origin excluded, and 1/*x* weighting using TargetLynx (MassLynx 4.2 software, Waters) and then used to calculate dTG and dC concentrations in patient samples and controls.

**Table 1:** The specific mass spectrometric settings for each substance and their corresponding internal standard. MRM transitions with protonated molecule and monitored fragments in positive mode. Retention time, cone voltage, and collision energy for each analyte

Substance	ESI mode	Retention time (min)	MRM transitions (m/z) Quantifier/qualifier	Cone voltage (V)	Collision energy (eV)
dTG	+	2.28	283.97>150.61 283.97>167.72	22 22	34 10
dTG-IS	+	1.72	283.00>143.94 283.00>161.96	4 4	34 10
dC	+	0.9	227.97>94.28 227.97>111.54	2 2	36 10
dC-IS	+	0.9	230.97>114.54	8	12

Abbreviations: *dTG*, 2′-deoxythioguanosine; *dTG-IS*, 2′-deoxyguanosine-^13^C_10_^15^N_5_; *dC*, 2′ deoxycytidine; *dC-IS*, 2′-deoxycytidine-^13^C_15_N_2_

### Method validation

#### Selectivity

Selectivity was assessed by analysing hydrolysed DNA from six different individuals not medicating with thiopurines, without the addition of internal standard or analytes for evaluation of interference from endogenous components. In addition, the above hydrolysed DNA samples were also prepared with internal standard working solution to evaluate possible analyte interferences. The absence of interferences was accepted as a response of less than 20% of the LLOQ for the analytes and 5% for the internal standards [24].

#### Matrix effect, extraction recovery, and process efficiency

Matrix effects were investigated using two different methods. First, qualitative matrix effects were explored. dTG, dC, dTG-IS, and dC-IS at a concentration of 50 nmol/L in 1 mmol/L DTT were infused at a flow rate of 10 µL/min post-column. Simultaneously with the post-column infusion, thiopurine-free hydrolysed DNA extracts without internal standard or analyte were injected (*n* =6) [25]. An increase or decrease of the continuous signal indicated ion enhancement or suppression. Possible interferences from phospholipids were measured by monitoring the mass transfer m/z 184→184 with injection of thiopurine-free hydrolysed DNA extracts [26]. Matrix effects were quantitatively evaluated according to Matuszewski et al. [27]. Matrix effect, extraction recovery, and process efficiency were calculated using three sets of samples with six samples in each set. dTG, dTG-IS, and dC-IS at concentration levels corresponding to calibration levels 0.04 (I) and 2.5 (VII) nmol/L for dTG, the internal standards, dTG-IS (1 nmol/L) and dC-IS (2.5 µmol/L) were either dissolved directly in 1 mmol/L DTT (set one), or added to DNA samples from thiopurine-free individuals either after (set 2) or before (set 3) the method procedure. All the samples were prepared according to the procedure previously described before analysis with LC-MS/MS. The matrix effect was defined as the mean peak areas of set 2 in relation to corresponding mean peak area of set 1, expressed as a percentage. A calculated value of 100% consequently implied that no matrix effects were apparent. Recovery and process efficiency were determined in a similar way by division of set 3 to set 2 and set 3 to set 1, respectively. The calculated matrix effect should fall within the interval of 85 to 115% and the extraction recovery should be more than 50% [28].

#### Calibration model

The calibration model was evaluated using the peak area ratio of each calibration standard versus corresponding IS, using TargetLynx. The appropriate regression model—linear or quadratic fit, without weighting or with 1/*x*—was determined by evaluating the size and distribution of the residual values for the calibrators as well as the coefficient of determination (*R*^2^). For each compound, a curve fit that resulted in approximately random residual distribution with concentration and a coefficient of determination (*R*^2^) > 0.995 defined the calibration function.

#### Lower limit of quantification

The lower limit of quantification (LLOQ) and limit of detection (LOD) were assessed by quantifying the lower calibration point (Std I); Std I was diluted 1:1 followed by one additional dilution 1:1 [29]. Six independent measurements of each dilution were analysed. Concentrations were calculated from the standard curve, followed by analyses of imprecision and inaccuracy. Acceptable precision and accuracy for LLOQ were set to ≤20% and 80–120%, respectively [24].

#### Accuracy and precision

The accuracy and precision of the method were determined through repeated measurements of QC samples at three levels (*n* =8). Intra-day and inter-day assay precision and accuracy were performed in one run and in multiple runs, respectively. Accuracy was calculated as a biased, mean measured concentration divided by spiked concentration × 100, and precision as the coefficient of variation (CV%). Mean inaccuracy should be within 15% (20% for LLOQ) of nominal values for quality control samples. The imprecision should not exceed 15% (20% for LLOQ).

#### Sample dilution

Aliquots of DNA at concentrations from 30 to 60 ng/µL were diluted with 1 mM DTT two times and reanalysed to evaluate the relationship between sample dilution, hydrolysis efficiency, and found concentrations of dTG and dC.

#### Carry-over

Carry-over was assessed by injecting ten sample extracts of the lowest calibration standard, followed by a series of injections alternating between the highest and lowest calibrator (*n* =10 per level). A *t*-test was used to compare the mean peak areas of the two sets of low calibration standards. Carry-over was considered insignificant if *p* >0.05 [30].

#### Stability

A system suitability check was used regularly before every batch confirming retention time, peak area, height, width, and the signal-to-noise ratio (*S*/*N*).

Stock solution and working solutions of dTG, dC, and internal standards stability were evaluated after 6 months’ storage at −20°C. Furthermore, the stability of hydrolysed DNA samples in the autosampler, calibrators, and controls was evaluated. The long-term storage stability of the patient samples as blood or DNA extract over 4 years at −80°C was evaluated. Storage stability was claimed if the bias between the freshly prepared samples and samples stored for 4 years ≤15%.

## Results and discussion

### Chromatography

**Fig. 3:**
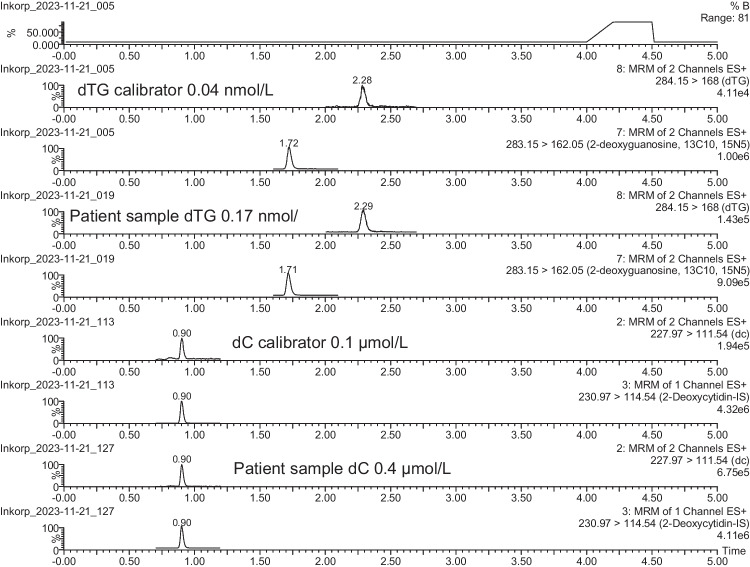
Representative MRM chromatograms from hydrolysed calibration standards and a patient sample. Chromatograms in the following order: (I) calibration standard of dTG 0.04 nmol/L and dTG-IS, (II) sample with 0.17 nmol/L dTG and dTG-IS, (III) calibration standard of dC 0.1 µmol/L and dC-IS, and (IV) sample with 0.4 µmol/L and dC-IS

For the chromatographic separation of the antimetabolite dTG and natural nucleosides, together with the internal standards, an HSS-T3 C18 column was found to be the most suitable. This UHPLC column gave extremely narrow and sharp peaks, leading to the highest possible sensitivity. This column utilises a trifunctional C18 alkyl phase covalently bound at a lower ligand density, leading to a stationary phase that is less hydrophobic and has better compatibility with aqueous mobile phases, thus promoting retention of polar compounds. The HSS T3 stationary phase, with its novel end-capping, provided good retention and selectivity for the natural nucleosides as well as for synthetic analogues with their generally high polarity. Figure 3 depicts representative MRM chromatograms from a hydrolysed calibration standard of 0.04 nmol/L dTG, and 0.1 µmol/L dC, together with a patient sample containing 0.17 nmol/L dTG, and 0.4 µmol/L dC, along with their corresponding internal standards.

### Selectivity

Samples from six thiopurine-free individuals were prepared for analysis in a two-step sample preparation process consisting of DNA extraction from whole blood followed by hydrolysis into nucleosides. The resulting analysis showed an absence of endogenous interferences at the mass transitions at the retention times for dTG, dC, dTG-IS, and dC-IS. Drug-free samples spiked with the internal standard showed no interfering peaks for dTG and dC.

### Matrix effect, extraction recovery, and process efficiency

The qualitative matrix effect experiment measuring ion suppression and enhancement showed no interferences in the chromatographic regions for dTG, dC, dTG-IS, and dC-IS. Finally, no interfering phospholipids were found at the retention times for dTG, dC, dTG-IS, and dC-IS. For evaluation of quantitative matrix effects, the mean extraction recovery, matrix effect, and process efficiency were calculated. The results are summarised in Table 2 for the concentration levels 0.04 and 2.5 nmol/L for dTG and the internal standard levels of 1 nmol/L for dTG-IS, and 2.5 µmol/L for dC-IS. Qualitative matrix effect could be calculated for dC-IS but not for dC, as the thiopurine-free DNA samples contain dC. For dTG, the extraction recovery for calibrators I and VII was 106.7% and 105.2%, with a matrix effect between 90.2 and 91.9% and a process efficiency within 94.8 to 98.0%. For the two internal standards, dTG-IS and dC-IS extraction recovery was within 102.2 to 107.2%, with a matrix effect of 86.7–93.8% and a process efficiency of 88.6–100.5%.

**Table 2:** Results of matrix effect (ME), extraction recovery (ER), and process efficacy (PE) for dTG, dTG-IS, and dC-IS determined at low and high concentrations, as mean values in six different hydrolysed DNA samples

*n* =6	Calibrator I			Calibrator VII		
	0.04 nmol/L			2.5 nmol/L		
Compound	ME%	ER%	PE%	ME%	ER%	PE%
dTG	91.9	106.7	98.0	90.2	105.2	94.8
	Internal standards					
dTG-IS	1 nmol/L					
	ME%	ER%	PE%	ME%	ER%	PE%
	91.2	106.0	96.7	86.7	102.2	88.6
dC-IS	2.5 µmol/L					
	ME%	ER%	PE%	ME%	ER%	PE%
	93.8	107.2	100.5	89.1	105.2	93.7

Abbreviations: *dTG*, 2′-deoxythioguanosine; *dTG-IS*, 2′-deoxyguanosine-^13^C_10_^15^N_5_; *dC-IS*, 2′-deoxycytidine-^13^C_15_N_2_

Finally, the quantitative matrix effect uncompensated for dTG and the internal standard normalised matrix factors were calculated. Absolute and compensated values for matrix effect and process efficiency as well as variability of matrix effect for the two different calibration levels are summarised in Table 3. For dTG uncompensated matrix effect, the CV ranged between 5.7 and 8.5%, and for dTG-IS’ compensated matrix effect, the CV ranged from 3.0 to 4.0%.

**Table 3:** Absolut and compensated (dTG-IS normalised) values for matrix effect (ME) and process efficiency (PE) for dTG, as mean values in six different hydrolysed DNA samples

*n* =6		Calibrator I				Calibrator VII			
		0.04 nmol/L				2.5 µmol/L			
Compound		ME%	CV%	PE%	CV%	ME%	CV%	PE%	CV%
dTG	Absolute	91.9	5.7	98.0	9.3	90.2	8.5	94.8	6.5
	Compensated	101.2	4.0	102.5	7.2	104.1	3.0	107.3	3.4

Abbreviations: *dTG*, 2′-deoxythioguanosine; *ME*, matrix effect; *PE*, process efficacy

### Calibration model, LOD, and LLOQ

A linear regression model, origin excluded, with 1/*x* weighting was used for both analytes. For dTG in the concentration range 0.04–2.5 nmol/L, resulted in coefficients of determination (*R*^2^) between 0.99586 and 0.99958 (*n* =6). For dC in the concentration range 0.1–12.5 µmol/l, resulted in coefficients of determination (*R*^2^) between 0.99586 and 0.99956 (*n* =6). The recalculated concentrations of each calibration standard were within ±2.3% for dTG and ±3.0% for dC. LOD was determined to a concentration of 0.003 nmol/L for dTG and 0.019 µmol/L for dC, and a LLOQ for dTG of 0.009 nmol/L and for dTG 0.064 µmol/L. dC values were calculated from the described sample process, including the final dilution.

### Accuracy and precision

Data on precision and accuracy of QC samples at three concentration levels for dTG and dC are summarised in Table 4. For dTG, intra-day imprecision was 3.2 to 4.5% and inter-day imprecision 9.2 to 9.5% and corresponding values for dC intra-day imprecision were 3.0 to 5.1%, and inter-day imprecision 8.4 to 10.9%. For dTG, the mean intra-day accuracy was 96.0 to 101.1% and inter-day accuracy 96.0 to 110.0%, and for dC, a mean intra-day accuracy was between 105.3 and 108.0% and inter-day accuracy from 98.5 to 105.3%. The use of a freshly prepared daily mix of the EpiQuik reagents and internal standard decreased the variation of added internal standard from a CV of 6.2 to 1.3% (*n* =5) (data not shown).

**Table 4:** Accuracy and precision for the quality controls for all included substances. From a single run on eight replicates at each quality control level (inter-day assay) and from eight different days (intra-day assay)

		Intra-day assay (*n* =8)			Inter-day assay (*n* =8)		
Substance		QC I	QC II	QC III	QC I	QC II	QC III
		0.1 nmol/L	0.5 nmol/L	3.75 nmol/L	0.1 nmol/L	0.5 nmol/L	3.75 nmol/L
dTG	Mean SD Imprecision (CV%) Accuracy (%)	0.10 0.005 4.5 101.1	0.05 0.016 3.2 99.2	3.6 0.1 4.1 96.0	0.11 0.01 9.2 110.0	0.48 0.04 9.2 96.0	3.8 0.36 9.5 101.3
		QC I	QC II	QC III	QC I	QC II	QC III
		0.25 µmol/L	1.3µmol/L	9.4 µmol/L	0.25 µmol/L	1.3 µmol/L	9.4 µmol/L
dC	Mean SD Imprecision (CV%) Accuracy (%)	0.27 0.01 5.1 108.0	1.37 0.04 3.0 105.4	9.9 0.48 4.9 105.3	0.25 0.03 10.9 100.0	1.28 0.11 8.4 98.5	9.9 0.9 9.1 105.3

Abbreviations: *dTG*, 2′-deoxythioguanosine; *dC*, 2′-deoxycytidine; *QC*, quality control; *CV*, coefficient of variation. Mean and SD in nmol/L for dTG and µmol/L for dC

### Sample dilution

Six different patient samples were diluted 1:1, with 1 mmol/L DTT, followed by one further 1:1 dilution. The diluted samples were back-calculated to undiluted concentrations whereupon. For the dTG (nmol/L) concentration for the six samples, the coefficient of variation was between 2.9 and 12.9%, and for dC (µmol/L) between 1.0 and 9.7%, and the ratio moles dTG/10^6^ moles dC was between 4.1 and 10.1% (see Table 5). The DNA concentration range obtained by dilution of these six samples reflects the interval obtained by processing the blood samples from study patients with Maxwell 16. Most samples processed resulted in a concentration range between 20 and 30 ng/µL DNA, indicating that the hydrolysis efficiency was maintained for the two analytes despite differing amounts of DNA in the samples.

**Table 5:** Sample dilution. Six samples were analysed undiluted, diluted 1:1 followed by one further dilution 1:1. The diluted sample concentrations were back-calculated as undiluted. For every sample (*n* =3), calculation of mean, standard deviation, and coefficient of variation for dTG in nmol/L, dC µmol/L, and ratio moles dTG/10^6^ moles dC was done

Patient	Amount of DNA ng/µL	dTG nmol/L Mean	Stdev	%CV	dC µmol/L Mean	Stdev	%CV	Moles dTG/10^6^ moles dC Mean	Stdev	%CV
1	27.8	1.8	0.24	12.9	1.4	0.17	9.7	1267.5	51.4	4.1
2	32.1	2.3	0.17	7.5	1.9	0.09	4.6	1237.1	54.4	4.4
3	44.0	2.3	0.07	2.9	2.0	0.16	7.8	1148.9	57.6	5.0
4	34.3	1.8	0.17	9.2	2.4	0.02	1.0	748.1	75.8	10.1
5	66.8	0.7	0.03	4.6	1.0	0.07	6.9	763.0	73.5	9.6
6	61.5	1.5	0.05	3.1	0.8	0.03	3.7	1884.3	42.9	2.3

Abbreviations: *dTG*, 2′-deoxythioguanosine; *dC*, 2′-deoxycytidin

### Carry-over

To prevent carry-over and ensure precision and accuracy, the washing procedure for the autosampler must be optimised. Our standard washing for the injection system with a weak wash solution containing 10:90 acetonitrile:water and a strong wash solution 80:20 acetonitrile:water was tested. Ten injections of the lowest calibrator followed by alternate injections of the highest and lowest calibrators were performed. Using the independent sample *t*-test to compare the mean of the two sets of the lowest calibration standards resulted in *p*-values above 0.05 for dTG, dTG-IS, dC, and dC-IS, with the conclusion that no carry-over was found.

### Stability

**Fig. 4:**
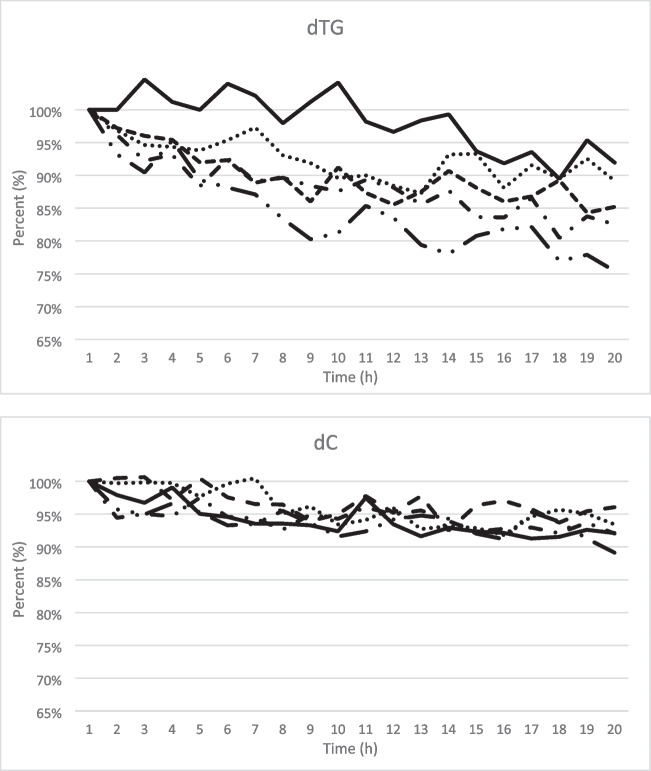
Summary of experiment to extend the stability of the samples in the autosampler. A combination of prolonged denaturation time after the hydrolysis and addition of formic acid gave an acceptable sample stability in the autosampler for up to 20 h. I. Hydrolysis for 1 h, followed by denaturation at 90 °C for 10 min, and finally cooling to 4 °C. II. Hydrolysis for 1 h, followed by denaturation at 90 °C for 10 min, and finally cooling to 4 °C. Adding formic acid to a final concentration of 0.1%. III. Hydrolysis for 1 h, followed by denaturation at 90 °C for 20 min, and finally cooling to 4 °C. IV. Hydrolysis for 1 h, followed by denaturation at 90 °C for 20 min, and finally cooling to 4 °C. Adding formic acid to a final concentration of 0.1%. IV. Hydrolysis for 1 h, followed by denaturation at 90 °C for 20 min, and finally cooling to 4 °C. Adding formic acid to a final concentration of 0.1%. V. Hydrolysis for 1 h, followed by denaturation at 90 °C for 20 min, and finally cooling to 4 °C. Adding formic acid to a final concentration of 0.5%.

Various tests were done to extend the stability of the samples in the autosampler. A combination of prolonged denaturation time after the hydrolysis and addition of formic acid gave an acceptable sample stability in the autosampler for up to 20 h. After hydrolysis for 1 h, the enzyme was denatured at 90°C for 20 min, and finally cooling to 4°C. Before loading the samples into the autosampler, formic acid was added to a final concentration of 0.5%. Above all the stability of dTG was improved (Fig. 4). DTT solutions are stable up to 3 months at −20°C (Data sheet Merck), which affects the stability of calibrators and controls for a maximum of 3 months. DTT must be present in the calibrator and controls solution to avoid dimerisation of the sulphur-containing dTG [31]. During the development process, we found that freeze/thaw of the calibrator and controls must be avoided (data not shown).

As samples should be collected from a cohort of about 500 patients over several years, proof of sample stability was required. To be able to draw conclusions from the measured concentrations of dTG and dC, the chemical stability of these analytes in two matrices, whole blood, and extracted DNAs, was investigated. Whole blood samples from nine patients and corresponding DNA, extracted at arrival to the laboratory, have been stored for up to 4 years at −80°C. After 4 years, DNA was extracted from the frozen whole blood and compared with DNA frozen for 4 years. In Table 6, the results from the nine patients reanalysed show no difference in concentration for dTG and dC nor the calculated dTG/dC ratio. The results from two-sided paired *t*-test were calculated for dTG, dC, and dTG/dC ratio and no significant difference was found between results from freshly extracted DNA or stored for 4 years.

**Table 6:** Comparison between DNA samples, extracted from whole blood at arrival to the laboratory, and stored 4 years at −80°C and DNA samples extracted from frozen whole blood. After 4 years, DNA was extracted from the frozen whole blood and compared with DNA frozen for 4 years. The results from two-sided paired *t*-test showed no significant difference between dTG, dC, and dTG/dC ratio values between day 0 and 4 years. %RE = (mean−mean day zero)/mean day zero × 100

	Day 0			Four years		
	dTG nmol/L	dC µmol/L	Ratio dTG/dC ×10^−3^	dTG nmol/L	dC µmol/L	Ratio dTG/dC ×10^−3^
Patient						
1A	0.09	1.0	0.09	0.10	0.9	0.11
2A	0.56	1.1	0.52	0.50	1.0	0.51
3A	0.38	0.8	0.47	0.39	0.8	0.49
4A	1.26	0.7	1.78	1.47	0.8	1.89
5A	0.73	0.9	0.77	1.13	1.3	0.90
6A	1.69	0.7	2.38	1.88	0.7	2.58
7A	0.93	1.4	0.68	0.77	1.2	0.65
8A	0.56	0.7	0.82	0.54	0.7	0.82
9A	1.24	1.0	1.20	1.20	1.0	1.26
Mean	0.83	0.9	0.97	0.89	0.9	1.02
%RE				7.3	−1.2	5.7

Abbreviations: *dTG*, 2′-deoxythioguanosine; *dC*, 2′-deoxycytidine

## Discussion

### Application of method

Samples from IBD patients, included in our ongoing study, on azathioprine therapy and in clinical remission for more than 6 months were analysed to evaluate the applicability of the method (see Table 7). In this group of 20 IBD patients, 5 were diagnosed with ulcerative colitis and the other 15 had been diagnosed with Crohn’s disease. The azathioprine dose among these patients varied between 25 and 275 mg/day and the dTG concentrations fell between 0.19 and 1.64 nmol/L, and for dC, an interval from 0.49 to 1.60 µmol/L was found. From the concentration data, a dTG/dC ratio was calculated with a variation between 0.14 × 10^−3^ and 1.57 × 10^−3^. To compare our data with earlier studies, we expressed the data as mol dTG/10^6^ mol dC, and using the measured DNA concentration, the quantity of dTG was expressed as fmol dTG/µg DNA. With the calculated range between 140.1 and 1573.8 dTG/10^6^ mol dC, the data are comparable with Coulthard et al. [20]. However, it must be noted that they measured dA instead of dC, and accordingly, their unit was dTG/10^6^ mol dA. Expressing the results as fmol dTG/µg DNA, the data range from 54.2 to 922.1 and comparing these data with the studies from Jacobson et al. and Coulthard et al. show similar results [19, 20].

**Table 7:** Quantification of incorporated dTG and dC from IBD patients included in our ongoing study, treated with azathioprine (dose) and in clinical remission for more than 6 months

Patient	Disease	Dose mg	dTG (nmol/L)	dC (µmol/L)	Ratio dTG/dC ×10^−3^	mol dTG/10^6^ mol dC	fmol dTG/µg DNA
1	UC	100	0.27	0.49	0.55	551.0	314.0
2	UC	25	0.24	0.64	0.37	369.6	196.7
3	CD	100	0.25	0.52	0.47	472.2	244.4
4	CD	200	1.11	0.91	1.22	1219.8	576.8
5	CD	150	1.28	1.44	0.89	886.4	922.1
6	CD	150	1.42	1.04	1.36	1364.3	639.7
7	CD	275	1.64	1.50	1.09	1090.5	647.6
8	CD	25*	0.15	0.83	0.18	184.1	112.1
9	CD	125	0.56	1.25	0.45	446.6	260.7
10	CD	75	1.36	0.86	1.57	1573.8	887.7
11	UC	100	0.39	0.46	0.84	842.8	378.4
12	CD	40*	0.19	1.36	0.14	140.1	54.2
13	CD	50	0.50	0.86	0.58	581.8	314.5
14	UC	100	0.55	1.60	0.34	344.6	217.6
15	UC	150	0.92	0.71	1.30	1301.8	631.4
16	CD	150	0.74	1.05	0.70	703.6	202.2
17	CD	100	0.91	1.10	0.82	821.6	514.3
18	CD	25	0.34	0.78	0.43	433.0	162.2
19	CD	100	0.69	1.00	0.69	693.5	533.0
20	CD	50/25*	1.01	0.77	1.31	1309.9	529.2

Abbreviations: *CD*, Crohn’s disease; *UC*, ulcerative colitis; *dTG*, 2′-deoxythioguanosine; *dC*, 2′-deoxycytidin. *Every other day

## Conclusions

The work presented describes a fully developed and validated method consisting of DNA isolation from whole blood, digesting of DNA into nucleotides, and measurement of the quantities of incorporated dTG and dC. The strategy during this method development has been to develop a high-throughput method at our laboratory, for routine setting, and for use on a weekly basis for measuring incorporated dTG in DNA. An analytical process where dTG and dC and their corresponding internal standard show/demonstrate high extraction/hydrolytic recovery minimised assay variation, and matrix effects. Using a chromatographic column with the HSS T3 stationary phase resulted in an optimal chromatographic performance, selectivity, and peak shape for the natural nucleosides and synthetic analogues.

The initial results, including 20 patient samples, analysed with the described method, and expressed in the unit fmol/µg DNA agree with earlier published studies [19, 20]. However, contrary to these methods, in our method, the complementary natural nucleotide dC is measured and considered a more correct measure if the dTG/dC ratio is calculated and used as a tool for interpreting results. The described method and sample processing will be used in an ongoing study of IBD patients to scrutinise whether measuring the pharmacological markers—incorporated dTG or ratio dTG/dC—is more accurate or a new component in the TDM toolkit to interpret clinical response for thiopurine-treated patients.
